# Scarring in Alcoholic Liver Disease: New Insights and Emerging Therapies

**Published:** 1997

**Authors:** Scott L. Friedman

**Affiliations:** Scott L. Friedman, M.D., is a professor of medicine and director of liver research at Mount Sinai School of Medicine, New York, New York

**Keywords:** chronic AODE (alcohol and other drug effects), alcoholic liver disorder, fibrosis, biological activation, cell and cell structure, cytokines, liver function, treatment method, literature review

## Abstract

Like other organs, the liver responds to injury (e.g., from chronic alcohol ingestion) with scar formation (i.e., fibrosis). Specialized cells known as stellate cells play a major role in the development of liver fibrosis. Normally these cells serve as important storage depots for vitamin A, but during alcoholic injury, a collection of cellular and molecular mediators cause stellate cells to undergo a process of activation that results in dramatic changes in their structure and function. Activated stellate cells then become primary producers of scar tissue. In turn, accumulated scar provokes a series of events that contributes to deteriorated liver function. An improved understanding of the factors that trigger stellate cell activation has led to new therapeutic approaches for reversing or preventing liver fibrosis more effectively.

Scarring (i.e., fibrosis) is the most severe consequence of liver injury from any cause, including injury caused by chronic alcohol ingestion. Although liver fibrosis is reversible, if it persists because of progressive alcoholic liver injury, irreversible cirrhosis will develop. Under this condition, the fibrous bands crisscrossing the liver contract, resulting in a severe distortion of the liver’s architecture that is characteristic of cirrhosis. As a consequence of the contraction, the liver’s size diminishes and its function is compromised, leading to impeded blood flow, reduced ability to detoxify drugs and foreign compounds, and impaired protein synthesis. (For a brief background on the function and anatomy of the normal liver, see sidebar, p. 311.) Collectively these changes can result in liver failure, often necessitating a liver transplant.

**Overview of Liver Structure and Function**

Occupying the upper right quadrant of the abdomen and extending into the upper left quadrant, the liver is one of the largest organs in the human body. Structurally, this 3½-pound organ consists of 50,000 to 100,000 functional units, called lobules, situated within an extensive network of blood vessels and bile ducts known as the portal triad (see figure). Each cylindrical lobule is typically several millimeters (mm) in length and up to 2 mm in diameter.

Small branches of the hepatic artery and portal vein running along each lobule’s outer edge supply blood, which flows into the lobule through tiny channels called sinusoids. Like spokes of a wheel, the sinusoids converge in the core of the lobule to form a central “drainage” vein or outflow region.

Sinusoids serve as the liver’s microcirculatory system. The endothelial cells composing the sinusoid walls contain large pore openings. These openings allow oxygenated blood from the hepatic artery and nutrient-laden blood flowing from the intestine via the portal vein to bathe the liver’s principal cells (i.e., hepatocytes), which are arranged in clusters between the sinusoids. Stacks of thin hepatocyte clusters just one or two cells thick (i.e., hepatic cellular plates) circle the lobule’s central vein (see figure).

Relatively large and metabolically very active, the hepatocytes are the workhorses that perform the varied and vital functions of the liver. These cells process and store nutrients, manufacture proteins, and remove toxins from the blood before it enters general circulation. Hepatocytes also produce bile, which collects between adjacent cells and drains into bile ducts for transport to the gallbladder. Thick, liquid bile stored in the gallbladder is released intermittently into the small intestine, where it aids in fat digestion.

**Figure:**
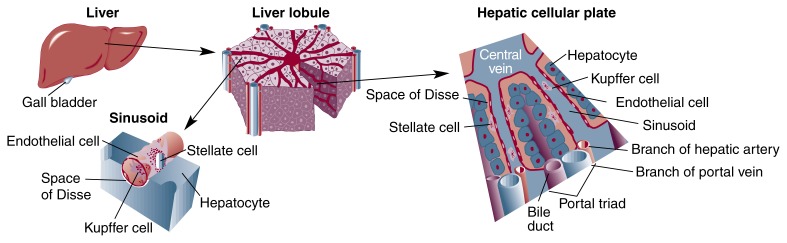
The liver is situated within the portal triad, an extensive network of blood vessels and bile ducts.

In addition to hepatocytes, several other types of cells with special functions are found in the liver. Kupffer cells, for example, are specialized immune cells lining the sinusoids. These highly efficient tissue scavengers (i.e., macrophages) remove more than 99 percent of the bacteria in the blood and can ingest a bacterium in less than 0.01 second. Because blood coming through the portal vein contains a substantial quantity of bacteria from the gastrointestinal tract, Kupffer cells are key players in disease prevention.

The sinusoidal endothelial cells have special functions, too. Not only do they form the wall lining of the sinusoids, but they also help clear circulating scar (i.e., extracellular matrix) molecules from the blood and are a potent producer of compounds that stimulate cell proliferation (i.e., growth factors).

Stellate cells (also known as lipocytes or as Ito, fatstoring, or perisinusoidal cells) play an important role in the liver as well. These star-shaped cells are found in the narrow space between the sinusoid wall and the hepatocytes (i.e., in the space of Disse). The cells contain numerous droplets of vitamin A, a nutrient essential for normal growth and vision. In fact, stellate cells are the body’s primary storehouse for vitamin A, and they store enough of this nutrient to supply the body’s needs for up to 10 months without any additional dietary intake. In fibrosis, they play a key role in producing scar protein.

—*Mary Beth de Ribeaux*

BibliographyGuytonACTextbook of Medical Physiology7th editionPhiladelphiaW.B. Saunders Co.1986

*Mary Beth de Ribeaux is a science editor of* Alcohol Health & Research World.

Currently, numerous liver transplants in the United States are performed in patients with end-stage alcoholic cirrhosis.^1^ However, the disease exacts a far larger economic and social toll in patients who are not candidates for a liver transplant (e.g., because of continued alcohol abuse; concurrent diseases, such as HIV [human immunodeficiency virus] infection, that would contraindicate a transplant; or advanced age) and who subsequently die of progressive liver disease.

During the past decade, exciting progress has been made in the understanding of how liver fibrosis (i.e., hepatic fibrosis) develops in patients with alcoholic liver disease (ALD). New methods of isolating scar-forming cells in animal and human livers and an improved understanding of how growth factors^2^ drive the liver’s response to injury have been key advances fueling this progress and have resulted in more realistic hopes for antifibrotic therapy in the future.

This article focuses on new insights about how the liver responds to injury by scar formation, the cell types and chemical messengers (i.e., cytokines, such as growth factors) that are involved, and how these insights point toward effective treatments.

## Liver Fibrosis: A Wound-Healing Response

The recognition that liver fibrosis represents a general wound-healing response has helped scientists understand the process of fibrosis development. The essential components of this response are a fibrogenic cell type, scar formation (i.e., modification of the normal matrix scaffolding in which cells are embedded), and growth factors. In the liver, the same fibrotic reaction occurs regardless of the type of injury, whether the damage results from alcohol, viral hepatitis, drugs toxic to the liver (e.g., methotrexate, a medication used to treat various types of cancer and arthritis), or immunologic or genetic liver disease. Moreover, the same general paradigm extends to tissues in other organs, including the kidney and lung. This broad view has allowed investigators studying different organ systems to pool their expertise in search of common features underlying tissue fibrosis. For example, in all forms of tissue fibrosis, the same general types of scar component molecules predominate. These molecules include certain types of collagen that manufacture threadlike fibrils (i.e., collagen types I and III); specialized compounds known as glycoproteins (e.g., fibronectin, laminin, and tenascin); and several types of a particular kind of glycoprotein (i.e., proteoglycans, such as dermatan and chondroitin sulfate, which are two constituents of connective tissue).

**Figure 1:**
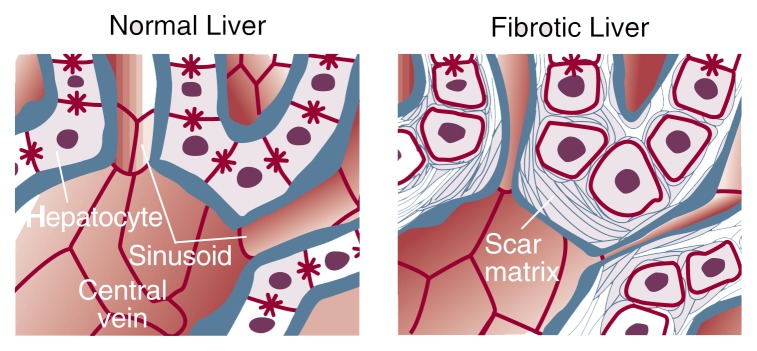
Pericentral fibrosis in alcoholic liver disease. Deposition of scar tissue around the hepatocytes near the central vein constricts blood flow through the sinusoidal channels.

Although the scar in alcoholic fibrosis has the same composition as the scar in other chronic liver diseases, its distribution is relatively unique. This distinctive distribution pattern is important in terms of both disease development (i.e., pathogenesis) and diagnosis. Early fibrosis around the outflow region of the sinusoid, near the central vein of the liver lobule (see figure 1), is a cardinal finding of ALD and portends a higher likelihood of progression to more severe fibrosis than when patients lack such pericentral fibrosis. In contrast, other forms of chronic liver disease are characterized by fibrosis in different sinusoid regions. In viral hepatitis, for example, fibrosis predominantly develops at the opposite end of the sinusoid, around its inlet or the portal triad.

The earliest site of injury preceding scar formation in ALD also occurs in the zone around the central vein. Thus, just as in other forms of liver injury, fibrosis in ALD tends to occur first where injury to cells (particularly hepatocytes) is greatest. Experimental evidence suggests that this early injury is attributable to impaired oxygen delivery (i.e., hypoxia) in the hepatocytes furthest from the source of oxygen-rich blood flowing from the hepatic artery through the portal triad. This so-called pericentral ischemia theory is a plausible explanation for injury in ALD. To begin with, two-thirds of the blood entering the liver is venous blood with low oxygen content. Although the remaining one-third of the blood supply normally delivers a sufficient amount of oxygen to the hepatocytes, alcohol metabolism increases their oxygen consumption as they break down alcohol to form acetaldehyde and then acetate.

Interestingly, fibrosis typically requires months to years of alcohol ingestion to develop; injury does not occur after a single binge of several days’ heavy intake, even though binges may lead to marked fat accumulation in hepatocytes and distortion of the normal sinusoidal architecture. This requirement for sustained injury also holds true for liver fibrosis associated with viral hepatitis and genetic liver disease. Although investigators do not know for certain why sustained injury is necessary before fibrosis develops, the ongoing production of scar tissue during chronic injury may eventually exceed the liver’s capacity to break it down through the secretion of specialized enzymes.

Only 10 to 15 percent of heavy drinkers develop alcoholic fibrosis. The risk of fibrosis, however, clearly increases with both longer duration of drinking and higher daily alcohol intake. Both genetic determinants and other cofactors affect the extent of this risk. Although genetic factors are not fully understood, they include gender (females are more susceptible than males) and possibly the specific type of immune response involved (i.e., which types of immune cells respond to the injury). In addition, other major cofactors in the development of alcoholic fibrosis include both hepatitis B and C viruses. Evidence for this link derives from several large, well-designed epidemiological studies that show a clear acceleration of liver fibrosis in patients with viral hepatitis who drink to excess. Most likely, the liver’s combined response to these two insults exceeds the severity of its response to either insult alone. However, a more direct interaction between alcohol (or its metabolites) and viral infection cannot be excluded.

**Figure 2:**
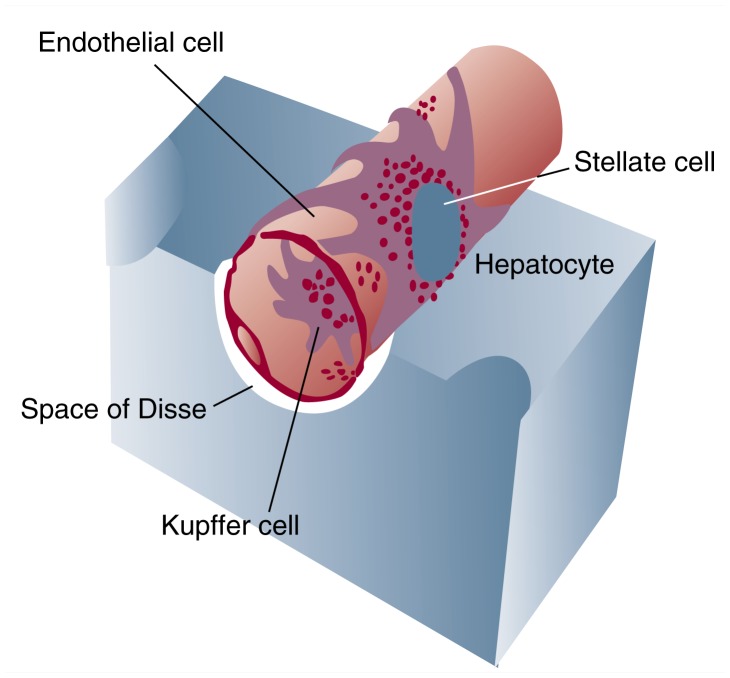
Cells of the hepatic sinusoid.

The liver’s major fibrogenic cell type is the hepatic stellate cell (see figure 2). These cells reside in the space between the hepatocytes and the sinusoidal endothelial cells (i.e., in the subendothelial space of Disse). The stellate cells are distributed throughout the liver, but they accumulate rapidly in areas of injury, as discussed in the following section.

In the normal liver, stellate cells are the main storage site for excess vitamin A compounds (i.e., retinoids), primarily droplets in the form of retinol bound to fatty acids (i.e., retinyl esters, such as retinyl palmitate). The stellate cell’s role as a depot for retinoids allows the body tight control over when and where vitamin A is delivered to tissues. Such control is an important protective mechanism, because vitamin A compounds can positively or negatively affect cell growth and differentiation, depending on the particular type of retinoid at work. Scientists do not clearly understand the role that vitamin A plays in the stellate cell’s response to injury and fibrogenesis, but future exploration of this question could lead to new insights into the pathogenesis of fibrosis.

## Stellate Cell Activation: The Central Event in Liver Injury

Among the most important advances in alcoholic fibrosis research has been the recognition that during alcoholic injury, stellate cells undergo a change in cellular behavior known as activation. Activation connotes a conversion from a resting stellate cell rich in vitamin A droplets to one that has lost most of its vitamin A droplets and is multiplying, producing large amounts of scar, and restricting blood flow by constricting the sinusoid. Current efforts to uncover the basis for alcoholic fibrosis focus on gaining an understanding of stellate cell activation.

**Figure 3:**
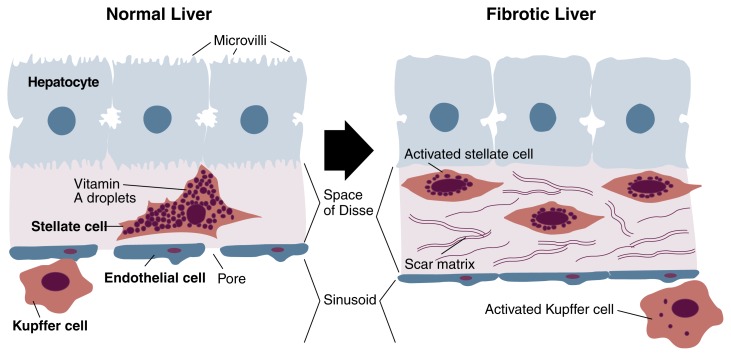
Progression of liver injury in alcoholic fibrosis. When stellate cells become activated, they secrete large amounts of scar tissue and lose the majority of their vitamin A droplets. Accumulated scar tissue causes hepatoctyes to lose their fingerlike projections (i.e., microvilli) and interferes with their normal function; it also causes endothelial cells to become less porous, which leads to impaired exchange of nutrients and other molecules to and from the sinusoids. Activation of Kupffer cells by alcohol may be responsible for setting off this chain of events. NOTE: Artist’s conception; not drawn to scale.

In progressive liver injury, stellate cell activation and the subsequent deposition of scar matrix in the space of Disse provoke a cascade of events that contribute to deteriorated liver function (see figure 3). This process has been referred to as “capillarization” of the sinusoid. The replacement of a normal, low-density matrix scaffolding in the space of Disse with a high-density scar matrix results both from disruption of the normal matrix by the release of matrix-degrading enzymes and from increased production of scar-forming proteins. These changes in subendothelial space composition create a microenvironment hostile to normal hepatocyte activity. Interaction between receptors on the hepatocytes’ cellular surface and the surrounding scar molecules probably mediates hepatocyte dysfunction. As a result, hepatocytes lose their small projections of cell membrane (i.e., microvilli) and deteriorate in their ability to perform key functions, including clearing toxins, secreting bile, and producing clotting proteins and albumin (a major protein of hepatocytes that is important for binding drugs and other compounds).

The altered composition of the subendothelial space also impairs the function of the endothelial cells lining the sinusoids. The pores (i.e., fenestrae) of these cells normally facilitate the transport of solutes from the blood to the hepatocytes. With scar accumulation, however, sinusoidal endothelial cells lose their porosity, and this loss hinders the exchange of large molecules and nutrients across the subendothelial space. Thus, scar accumulation in the space of Disse could lead to impaired breakdown of toxic drugs, for example.

Another key event within the sinusoidal milieu is the activation of the specialized immune cells that reside in the liver: macrophages known as Kupffer cells. Like other macrophages, Kupffer cells perform an important function by engulfing invading microorganisms and foreign compounds. In addition, they secrete growth factors and generate chemically unstable oxygen compounds (i.e., oxygen radicals) that are intended to fight infection by destroying microorganisms, such as bacteria and viruses, but also may have unwanted effects on neighboring cells. Increasingly, experimental work is focusing on how alcohol consumption leads to Kupffer cell activation, because the secretion of growth factors by activated Kupffer cells in turn may be a key stimulus to activating stellate cells. Many investigators believe that the activation of Kupffer cells in alcoholic liver injury results from the combined effects of alcohol and low doses of endotoxin, a product of gut-derived bacteria. Experimental animal models of alcohol feeding in rodents and primates have provided data supporting this view, and current efforts seek to identify the molecular mechanisms of Kupffer cell activation in these experimental settings.

## Cellular and Molecular Features of Stellate Cell Activation

**Figure 4:**
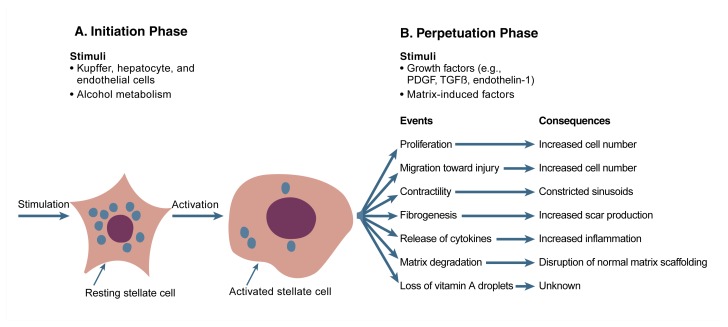
Phases of stellate cell activation. (A) Initiating factors from neighboring Kupffer cells, endothelial cells, and hepatocytes stimulate stellate cells to undergo the changes involved in activation. Alcohol may induce some of these initiating factors (e.g., compounds generated by alcohol-damaged hepatocytes); in addition, alcohol may more directly contribute to stellate cell activation through its metabolism (e.g., by creating oxygen-deficient conditions and by forming acetaldehyde). (B) In the perpetuation phase, continued stimulation from growth factors and an altered matrix scaffolding in the space of Disse lead to a cycle of increasing fibrosis and related consequences. PDGF = platelet-derived growth factor. TGFβ = transforming growth factor beta.

Regardless of the initial cause of injury, the fundamental features of stellate cell activation appear to be similar. Broadly, the process can be viewed in two phases: initiation and perpetuation (see figure 4). Initiation refers to the earliest changes in the stellate cells that render them more responsive to growth factors. Perpetuation refers to the effects of these growth factors on stellate cell responses, including growth and scar production.

For the most part, the initiating factors that trigger the earliest changes in stellate cells come from neighboring cells. Damaged hepatocytes may be a major source of these factors, because as hepatocytes are injured, they generate unstable oxygen radicals that disrupt cell membranes and may directly stimulate stellate cells. Similarly, stellate cells can be stimulated by factors released from activated Kupffer cells and from sinusoidal endothelial cells. The sinusoidal endothelial cells can rapidly release a specific subtype of the scar molecule fibronectin, which is capable of stimulating stellate cells.

Once stellate cells are “primed” by such initiating factors, perpetuation ensues, ultimately leading to progressive fibrosis. Although perpetuation initially represents an effort to limit injury, this mechanism is a wound-healing response gone too far when it continues to the point that extensive fibrosis occurs. The process of perpetuation can be subdivided into at least seven distinct events that can occur simultaneously (see figure 4):

- Proliferation of stellate cells
- Directed migration of additional stellate cells toward the injury (i.e., chemotaxis)
- Contractility, which allows cells to constrict the sinusoids and reduce blood flow, thus elevating pressure within the blood vessels entering the liver (i.e., creating portal hypertension, a major clinical problem in alcoholic cirrhosis)
- Production of more scar by each cell (i.e., fibrogenesis)
- Release of cytokines that increase the accumulation of inflammatory cells (i.e., white blood cells), which can disrupt the liver’s normal matrix scaffolding and further stimulate stellate cell activation
- Degradation of the liver’s normal matrix, which is required to preserve liver function
- Loss of vitamin A droplets, whose functional role is unclear.

## Alcohol’s Role in Stellate Cell Activation

Alcohol may contribute to stellate cell activation in several ways, as follows:

- Recent studies have noted that the activity of a particular series of transcription factors (i.e., proteins that regulate gene expression) increases specifically in response to an oxygen deficiency (i.e., hypoxia) in the hepatocytes. Because factors similar to the proteins identified in these studies are found in stellate cells as well as in other cells that are sensitive to reduced oxygen levels, researchers have speculated that hypoxia induced by alcohol metabolism may be a direct stimulus to stellate cells.
- The damage to hepatocytes that occurs in alcoholic hepatitis liberates chemically unstable compounds known as lipid peroxides and reactive oxygen intermediates. These compounds are increasingly recognized as fibrogenic mediators toward stellate cells.
- Like lipid peroxides and reactive oxygen intermediates, acetaldehyde, the first product of alcohol metabolism, also has some fibrogenic activity toward stellate cells. By itself, however, acetaldehyde does not provoke the entire fibrogenic cascade.
- Alcohol combined with small amounts of either whole bacteria or bacterial proteins from the digestive tract may activate Kupffer cells, which in turn may accelerate stellate cell activation, as previously mentioned.
- Alcohol metabolism may provoke the production of chemicals that attract white blood cells, which may then accelerate the disruption of the liver’s normal matrix scaffolding and hasten its replacement by scar matrix.

An ongoing controversy centers on whether inflammation is required for alcoholic fibrosis to develop. Settling this controversy has important implications, because if inflammation is always present, then medications that reduce inflammation could be effective in slowing fibrosis. Investigators who agree that inflammation is required argue that this condition is present biochemically even when not detectable in liver tissue examined under the microscope (i.e., inflammatory mediators and cytokines are active even though white blood cell accumulation, a hallmark of inflammation, may be transient or too subtle to visualize). In contrast, researchers who argue against the necessity of inflammation in alcoholic fibrosis point to the primate models of alcoholic liver injury, in which only a few inflammatory cells appear despite progressive fibrosis. In either case, however, alcoholic fibrosis appears to develop when the liver’s normal equilibrium state is perturbed, leading to tissue damage and the release of inflammatory and/or fibrogenic mediators with or without observable white blood cells.

## Emerging Therapies for Alcoholic Fibrosis

Unquestionably, the most effective therapy for alcoholic fibrosis is the cessation of alcohol use. In many patients, even severe inflammation and fibrosis can reverse almost completely with abstinence. In contrast, continued alcohol use in the presence of fibrosis virtually guarantees continued progression, often leading to cirrhosis with end-stage liver disease. It is unknown, however, when fibrosis reaches its irreversible point, either in terms of the amount of fibrosis present or the duration of disease.

Meanwhile, with the remarkable progress made in our understanding of the cellular basis of liver fibrosis, effective treatment to reduce scarring and its consequences (i.e., antifibrotic therapy) is moving closer to reality. Antifibrotic therapy may be directed toward one of several goals, including the following: inhibiting stellate cell activation, neutralizating the chemical messengers (i.e., cytokines) active during stellate cell activation, and preventing healthy matrix degradation while accelerating scar matrix degradation. Possible treatment options to accomplish each of these goals are discussed next.

### Inhibition of Stellate Cell Activation

Given the activated stellate cell’s primary role in almost every feature of fibrosis, reining in its activation is an attractive target. In pursuit of this goal, antioxidant therapy (e.g., vitamin E) is used to reduce the generation of oxygen radicals that can injure cells and set off stellate cell activation. Antioxidant therapy is already undergoing clinical trials in patients with chronic hepatitis C virus infection or alcoholic fibrosis and shows early promise. In addition, work by Lieber and colleagues (1994) has uncovered a potential benefit of the natural compound polyunsaturated phosphatidylcholine (PPC) in both primate experiments and pilot studies in patients with alcoholic fibrosis. A large multicenter trial is currently under way. Although the mechanism of PPC is not clearly established, it may function by stabilizing the alcohol-associated damage to cell membranes or by increasing the production of collagenase, an enzyme that breaks down scar matrix.

Two glycoproteins produced by white blood cells in response to viral infection (i.e., alpha and gamma interferon) directly slow stellate cell activation through unknown mechanisms. Their clinical use, however, has been restricted to patients with viral, rather than alcoholic, fibrosis, because these compounds can potentially modulate immune activity.

### Neutralization of Cytokines

Several chemical messengers contribute to the behavior of activated stellate cells. These cytokines include platelet-derived growth factor, transforming growth factor beta, and endothelin-1, which are produced by both stellate cells as well as surrounding Kupffer and endothelial cells. Currently, many pharmaceutical companies are testing compounds that neutralize these and related cytokines by either sequestering the cytokines or interfering with their ability to interact with their receptors. The key to realizing success with such compounds will be to target their activity to stellate cells and to limit unwanted consequences of cytokine inhibition apart from their activity in fibrosis.

In preceding decades, the antigout medication colchicine drew enthusiastic attention as an antifibrotic agent, and a single long-term trial demonstrated increased survival in a small number of cirrhotic patients who received this medication compared with patients who received a placebo. Although the drug is well tolerated, its exact mechanism of action is unknown, and no additional trials have corroborated its initial success. Further study is required to explore colchicine’s possible effect on stellate cells.

### Preservation of Healthy Matrix

Given that disruption of the liver’s normal matrix is a key early event in alcoholic fibrosis, efforts to inhibit the enzyme responsible for this disruption are under way. In addition, as scar matrix accumulates, its resorption also will be an important goal of therapy. Because different enzymes degrade healthy matrix and scar matrix, it may be possible in principle to develop inhibitors that could selectively block only the enzymes that disrupt the normal matrix and not those enzymes required for scar breakdown. The continued identification of specific enzymes, their cellular sources, and points of regulation will be essential steps in developing therapies that reduce fibrosis by affecting matrix degradation.

## Footnotes

1 In the United States, alcoholic cirrhosis accounted for 16.8 percent of the liver transplants performed from October 1, 1987, through December 31, 1993 (Evans 1997).

2 For a definition of this and other technical terms used in this article, see glossary, p. 330.
